# Implementation of peer support for people with severe mental health conditions in high-, middle- and low-income-countries: a theory of change approach

**DOI:** 10.1186/s12913-024-10990-5

**Published:** 2024-04-18

**Authors:** Ramona Hiltensperger, Grace Ryan, Inbar Adler Ben-Dor, Ashleigh Charles, Ellen Epple, Jasmine Kalha, Palak Korde, Yasuhiro Kotera, Richard Mpango, Galia Moran, Annabel Sandra Mueller-Stierlin, Rebecca Nixdorf, Mary Ramesh, Donat Shamba, Mike Slade, Bernd Puschner, Juliet Nakku

**Affiliations:** 1 Department of Psychiatry II, Ulm University, Ulm, Germany; 2 Centre for Global Mental Health, London School of Hygiene and Tropical Medicine, London, UK; 3 Department of Social Work, Ben Gurion University of the Negev, Be’er Sheva, Israel; 4 School of Health Sciences, Institute of Mental Health, University of Nottingham, Nottingham, UK; 5 Centre for Mental Health Law and Policy, Indian Law Society, Pune, India; 6 Butabika National Referral Hospital, Kampala, Uganda; 7 Department of Mental Health, School of Health Sciences, Soroti University, Soroti, Uganda; 8 Department of Psychiatry and Psychotherapy, University Medical Center Hamburg-Eppendorf, Hamburg, Germany; 9 Department of Health Systems Impact Evaluation and Policy, Ifakara Health Institute, Dar Es Salaam, Tanzania; 10 Health and Community Participation Division, Nord University, Namsos, Norway

**Keywords:** Peer support, Theory of change, Global mental health, Complex interventions, Implementation

## Abstract

**Background:**

Stakeholder engagement is essential to the design, implementation and evaluation of complex mental health interventions like peer support. Theory of Change (ToC) is commonly used in global health research to help structure and promote stakeholder engagement throughout the project cycle. Stakeholder insights are especially important in the context of a multi-site trial, in which an intervention may need to be adapted for implementation across very different settings while maintaining fidelity to a core model. This paper describes the development of a ToC for a peer support intervention to be delivered to people with severe mental health conditions in five countries as part of the UPSIDES trial.

**Methods:**

One hundred thirty-four stakeholders from diverse backgrounds participated in a total of 17 workshops carried out at six UPSIDES implementing sites across high-, middle- and low-income settings (one site each in India, Israel, Uganda and Tanzania; two sites in Germany). The initial ToC maps created by stakeholders at each site were integrated into a cross-site ToC map, which was then revised to incorporate additional insights from the academic literature and updated iteratively through multiple rounds of feedback provided by the implementers.

**Results:**

The final ToC map divides the implementation of the UPSIDES peer support intervention into three main stages: preparation, implementation, and sustainability. The map also identifies three levels of actors involved in peer support: individuals (service users and peer support workers), organisations (and their staff members), and the public. In the UPSIDES trial, the ToC map proved especially helpful in characterising and distinguishing between (a) common features of peer support, (b) shared approaches to implementation and (c) informing adaptations to peer support or implementation to account for contextual differences.

**Conclusions:**

UPSIDES is the first project to develop a multi-national ToC for a mental health peer support intervention. Stakeholder engagement in the ToC process helped to improve the cultural and contextual appropriateness of a complex intervention and ensure equivalence across sites for the purposes of a multi-site trial. It may serve as a blueprint for implementing similar interventions with a focus on recovery and social inclusion among people with mental ill-health across diverse settings.

**Trial registration:**

ISRCTN26008944 (Registration Date: 30/10/2019).

**Supplementary Information:**

The online version contains supplementary material available at 10.1186/s12913-024-10990-5.

## Background

Peer support is a complex mental health intervention in which people with lived experience of mental health conditions support others in their recovery [1]. Peer support is an established intervention in many high-income countries (HICs) [2, 3], and has been rapidly spreading to other parts of the world [4, 5]. Peer workers are employed in a variety of roles, for example in the provision of one-to-one support, facilitation of mutual support groups, or running mental health organisations and programmes [2, 6]. In the following, we refer to peer support workers as persons in recovery from a serious mental health condition who are hired to offer services to others with serious mental health conditions individually and in groups.

One of the biggest challenges in implementing peer support is to provide the appropriate conditions for it to succeed and be sustained [7]. Key stakeholders can offer important insights into these conditions, how they might be improved, or indeed how the intervention itself may need to be adapted to accommodate them. At the same time, engagement of local stakeholders can help to increase buy-in and pave the way for more successful implementation [8]. While the benefits of stakeholder (and more specifically, service user) engagement are well-documented by mental health research studies from HICs [9], there is less research on the subject in low- and middle-income countries (LMICs) [8]. However, in the field of global mental health, Theory of Change (ToC) is well-recognised as a useful tool for engaging stakeholders in the design, implementation and evaluation of complex mental health interventions, including peer support [10–13].

ToC is a theory-driven method that seeks to understand how and why an intervention or programme works [14]. ToC is increasingly used for planning, implementing, and evaluating complex interventions [15]. In order to benefit from different forms of expertise, a ToC map is often developed in a participatory way, bringing together a range of stakeholders such as service users, health service planners, health professionals. Through different forms of group communication (e.g., workshops, interviews) knowledge exchange between researchers and stakeholders takes place. This knowledge is then integrated into a ToC map [16]. The map represents an explicit theory of how a programme will achieve short-term and intermediate outcomes on its way to impact and visualizes those hypothesized steps along causal pathways in the local context. Table 1 describes core components of ToC (adapted from De Silva et al. [16]) using a worked example of a recovery-oriented training programme for mental health professionals in a large city aimed at improving well-being of service users.

**Table 1:** Core components of Theory of Change (ToC, adapted from De Silva et al. [16])

ToC Terminology	Description	*Example*
Impact and ceiling of accountability	The impact is the ultimate outcome or goal the project seeks to achieve. The impact is delineated from the long-term outcome by the ceiling of accountability. After this point, the intervention on its own is no longer responsible for achieving the stated impact.	*Improved well-being of people with mental health conditions in the metropolitan area*
Outcomes	Outcomes (short-term, intermediate or long-term) are the intended results of the intervention that need to occur in order to achieve the intended impact. They are connected via logical causal pathways (indicated in the ToC map by arrows). The final or long-term outcome is the last one the programme is able to influence on its own before reaching the ceiling of accountability.	*(Short-term) Sufficient number of mental health professionals recruited to cover the metropolitan area* *(Intermediate) Improvement in knowledge, attitudes and practices related to recovery among mental health professionals in the metropolitan area* *(Long-term) Improvement in well-being scores of people with mental health conditions receiving professional care in the metropolitan area*
Interventions	These are the different components of the complex intervention.	*Training for mental health professionals on recovery*
Assumptions	Assumptions describe external conditions that must exist for the outcome to be achieved but lie beyond the control of the project.	*Mental health professionals are sufficiently motivated to actively participate in training.*
Indicators	Indicators are a targeted way of measuring each outcome in order to verify whether or not it has been achieved.	*Mean difference in pre- and post-training scores among participating mental health professionals*
Rationales	Rationales provide explanation for why one outcome follows another or why certain actions must be taken to achieve the desired outcome. They may be based on evidence or experience.	*(Experience) Mental health professionals have identified a lack of training as one of the main barriers to delivering recovery-oriented care.* *(Evidence) Randomised controlled trials have shown recovery-oriented care to be effective in improving well-being, when compared to usual care provided by mental health professionals.*

The ToC is modified throughout the whole implementation and evaluation of the programme, allowing for a continual process of reflection on how change happens [16]. The ToC approach has been proven to be practical and feasible in high-, middle- and low-income settings [15].

One of the first records of using ToC across several sites in LMICs comes from the PRIME research consortium [10] which developed mental health care plans to integrate mental health into primary care in Ethiopia, India, Nepal, South Africa and Uganda. A cross-country ToC map was adapted in ToC workshops at each site to develop site-specific ToC maps. Also, ToC has previously been employed to scale-up an evidence-based psychological intervention for Syrian refugees to several sites in Turkey [17]. In both ToC studies cited above, the ToCs were developed prior to implementation or scale-up. However, ToCs may also be refined as a project evolves, as illustrated by the Future Health Systems consortium’s multinational study in Bangladesh, India and Uganda [12]. The consortium describes learnings from developing separate ToCs for each study site and revising these two years after the start of the project. Revision and reflection were considered crucial to adapt to changes in the outer setting of implementation. Taken together, ToC is now increasingly being used by multinational consortia to address the particular challenges multi-site implementation presents.

The study at hand contributes a further example of the use of ToC in a multisite trial supporting collaborative, transnational work. ToC was an essential part of the international UPSIDES project [18, 19] that scaled-up peer support interventions for people with severe mental health conditions at six study sites in a range of high-, middle- and low-resource settings. Conducting such a trial presents several challenges in the implementation of the intervention, such as engaging a very diverse range of stakeholders across all sites throughout the project, as well as the need to ensure fidelity to a core model while allowing for adaptation to different contexts. This paper describes the process by which local and cross-site ToC maps were developed within the UPSIDES project and revised over the course of the study, to provide general guidance for implementing peer support which can be applied in a variety of contexts, and to identify necessary local adaptations. It contributes to the growing literature on the application of ToC by international research consortia to help facilitate collaborative, transnational work and multi-site implementation.

## Methods

### Setting

The ToC maps were developed to guide the implementation and evaluation of the “Using Peer Support In Developing Empowering mental health Services (UPSIDES)” study [18, 19] carried out at: 1) Ulm University’s Department of Psychiatry and Psychotherapy II in Günzburg (UUlm), Ulm, Germany (high income), 2) University Medical Centre Hamburg-Eppendorf and city-wide community services (UKE), Hamburg, Germany (high income), 3) Butabika National Referral Hospital (BU), Kampala, Uganda (low income), 4) Muhimbili National Hospital at the Department of Psychiatry and Mental Health (DS), Dar es Salaam, Tanzania (formerly low income, now lower-middle income), 5) two community rehabilitation organisations (“Kidum Proyektim Shikumiim” and “Enosh”) which provide services across the country, coordinated by Ben Gurion University (BGU), Be’er Sheva, Israel (high income), and 6) Hospital for Mental Health in Ahmedabad, Gujarat, India, coordinated by the Centre for Mental Health Law and Policy (PU), Pune, India (lower-middle income). The study sites varied in the types of services offered (including inpatient, outpatient, or community services), previous experiences with peer support, and organisational readiness prior to the implementation of the UPSIDES intervention. A detailed description of intervention development in the UPSIDES study is reported elsewhere [20, 21]. More details on the overall UPSIDES research project are provided in other previous publications of the UPSIDES study group [7, 19, 22].

### Procedures

**Fig. 1:**
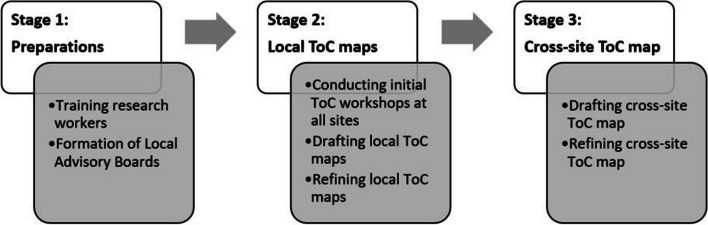
Cross-site ToC development process in three stages

The development of the cross-site ToC map was achieved in three stages (Fig. 1).

In Stage 1 (preparations), research workers at all study sites were trained by an UPSIDES consortium member (GR) with expertise in the use of ToC. Then, each UPSIDES study site established a Local Advisory Board (LAB) composed of local stakeholders. LAB members were purposively selected to represent diverse groups in line with guidance provided to all sites by the UPSIDES Implementation Plan [23], including: service user and carer representatives, clinical staff members, hospital directors and/or ministry of health representatives, local community leaders, traditional healers and/or religious leaders, as appropriate.

In Stage 2 (local ToC maps) each site conducted initial ToC workshops with LAB members between the end of 2018 and 2019. Facilitators’ guidance was provided in the UPSIDES Implementation Plan (see Appendix 6 on ToC workshops in the UPSIDES implementation manual [23], provided here as Supplementary file 1). The aims of these initial ToC workshops were to review the findings of the current stage analysis [7] and to develop a ToC tailored to the sites’ local circumstances to guide the development, implementation, and evaluation of the peer support intervention. Based on these initial workshops, the first drafts for local ToC maps were developed. These were then refined iteratively over the course of the project with additional workshops as needed throughout 2020 and 2021. During the lockdowns resulting from the COVID-19 pandemic some of these additional workshops were held online. Over the course of this process, the two German study sites developed a joint German ToC map for the implementation of UPSIDES peer support in Germany [24] which was used along with the other site-specific ToC maps for the development of the cross-site ToC. Workshops were held either in the local language or in English, depending on LAB members’ preferences. Bilingual research staff translated the local maps into English language where necessary, following a proportionate translation methodology which was developed as part of the UPSIDES project [25].

In Stage 3 (cross-site ToC map), a first draft for a cross-site ToC was drafted by RH and EE. The draft was then refined through several rounds of expert consultation with representatives of all sites and by all co-authors of this manuscript. In line with Breuer et al.’s Checklist for Reporting Theory of Change [15], this paper will describe the impact and long-term outcomes, the anticipated short- and medium-term outcomes along the process of change, the intervention components which happen at different stages of the pathway, assumptions about how change would occur, and additional ToC elements such as indicators, supporting research evidence, actors in the context, sphere of influence and timelines.

### Participants

LAB members were invited to participate in the ToC workshops at each study site. As described above, sites were provided standardised guidance on the selection of LAB members. There was a wide variety in the types of stakeholders who ultimately joined LABs and participated in ToC workshops. These ranged from service user and carer representatives, to outpatient and community mental health care staff, clergy and spiritual healers (Table 2). Sites were instructed to conduct ToC workshops with of 5–15 stakeholders of the LABs with complementary expertise. The initial workshops were followed by additional site-specific workshops where needed. These additional workshops could be held for one of two possible reasons: 1) they were held with new participants representing other types of stakeholders to complement and update the ToC maps with additional perspectives, or 2) they were held in separate workshops tailored to specific stakeholder groups, with participants who would otherwise have struggled to participate more confidently and actively due to power imbalance within mental health care services.

**Table 2:** Summary of data collection

Data source	Purpose
Paper-based (sticky notes, flipchart)	Site-specific ToC draft
Audio recordings of the workshops	Site-specific ToC draft
Meeting minutes	Site-specific ToC draft
Excel file	Synthesis of all elements for cross-site ToC
PowerPoint file	Graphical design of cross-site ToC

### Collection and synthesis of ToC data

The information used to develop the ToC maps was gathered through several workshops where key stakeholders came together to discuss what the ultimate impact of peer support will be, and then work backwards from that point to identify the key steps needed to bring about the previously identified impact. The workshops were led by facilitators who were researchers of the UPSIDES study consortium previously trained in conducting ToC workshops. To kick off the discussion, a facilitator asked the group a series of questions, such as: What real-world impact or change do we want to achieve with peer support? What outcomes are needed to achieve this impact? What interventions are needed to achieve these outcomes? As participants responded to these questions, the facilitator summarized their answers on sticky notes or loose sheets of paper and arranged them on a wall, large table, flip chart, or on the floor to provide a sense of the series of steps on the way to achieve the ultimate outcome. Some sites further collected information in the form of recordings and meeting minutes during the ToC workshops. All these sources of information were then further integrated into site-specific individual ToC maps by the researchers at each study site. Once all sites had produced their ToC maps, the elements of the site-specific maps were aggregated, compared, and categorized in Microsoft Excel to facilitate harmonisation into one cross-site ToC map. Aspects that were mentioned by many stakeholders and continued to be rated as important throughout the course of the study in several feedback rounds were prioritised for inclusion in the cross-site ToC map. The final cross-site ToC map was constructed using Microsoft PowerPoint. Table 2 provides an overview of all data sources and for what purpose they were collected.

## Results

A total of 17 workshops were held. Across all sites and workshops a total of 134 stakeholders from various backgrounds participated. Table 3 shows more details on the workshops and their participants.

**Table 3:** Number of participants at ToC workshops

Study site	UULM	UKE	BU	DS	BGU	PU	Total^e^
Number of workshops	5	4	1	2	4	1	17
Psychiatric staff^a^	6	2	3	10	-	1	22
Social/Community staff^b^	2	-	-	-	65	-	67
Lived experience^c^	7	8	2	6	4	1	28
Carers	2	-	1	-	-	-	3
Researchers	-	2	1	2	4	-	9
Other^d^	2	-	-	-	1	2	5
Total^e^	19	12	7	18	74	4	134

^a^Psychiatric hospital staff

^b^Social-psychiatric, social facility or community service staff

^c^Peer support workers or service user representatives

^d^Traditional healers, religious leaders, other community leaders

^e^Total numbers of individual participants per site and per type of participant given. Several participants have participated in more than one workshop

**Fig. 2:**
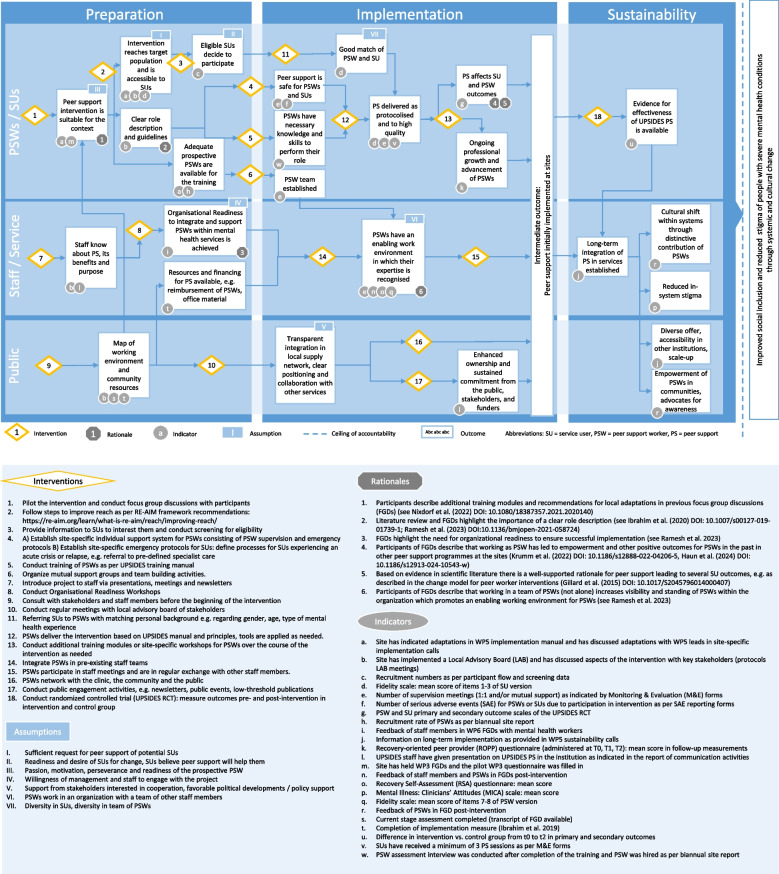
UPSIDES cross-site ToC map

The initial drafts of site-specific ToC maps along with an overview of main commonalities and differences between the initial drafts are provided in Supplementary file 2. The drafts show various stages of elaboration up to this point in January 2020, when they were first submitted by the sites. All sites defined several outcomes to be achieved during implementation and have specified rationales, interventions, indicators and assumptions to varying degrees. UULM, UKE and BU have defined a clear final impact statement. UKE, BU, DS and BGU have also already identified causal links between the ToC elements. Based on all these ToC drafts, the cross-site ToC map that harmonises insights from all six site-specific maps was developed. The final cross-site ToC map is presented in Fig. 2.

### Common elements across sites: the UPSIDES cross-site ToC map

The overall structure of the ToC map indicates a process over time to be read horizontally, with three main phases (from left to right, indicated by three columns): (1) preparation and pilot, (2) implementation, and (3) sustainability. Three main levels of actors are represented on the vertical axis from top to bottom: individuals (service users and peer support workers), organisations (and their staff members) and the public. The desired ultimate impact of the UPSIDES intervention to be achieved at all sites was identified through consensus as: “improved social inclusion and reduced stigma of people with severe mental health conditions through systemic and cultural change”.

#### Outcomes

The first step to achieve when preparing to implement UPSIDES peer support is to have a map of the working environment and community resources at the site in order to achieve suitable adaptation of the UPSIDES intervention to the local context. This is the precondition for several subsequent outcomes on different levels, namely: that the intervention reaches the target population and is easily accessible; there is a clear role description and guidelines for peer support workers (PSWs) specific to their local context; and staff members of the organisation in which the PSWs will be working know about the benefits and purposes of peer support. The next outcomes are the successful recruitment of service users (SUs) that are eligible and willing to participate, availability of prospective PSWs that match pre-defined criteria, as well as identification of staff members that will serve as allies for peer support in the organisation, promoting organisational readiness. Once these preparatory steps have been completed, practical implementation and the provision of peer support services can begin.

The next phase leads to the intermediate outcomes related to the initial implementation. In order to deliver peer support per protocol and to high quality, it is necessary to have trained PSWs in order for them to have the necessary knowledge and skills to perform their role. The establishment of a team of PSWs – rather than PSWs working on their own – is another important outcome along the way. Further, the site should establish mechanisms to ensure that peer support is safe for both PSWs and SUs and that SUs are appropriately matched to PSWs, considering diverse personal backgrounds. The next steps are to ensure that peer support is offered in a way that makes it empowering for both SUs and PSWs and ensures ongoing professional growth and advancement of PSWs. Also, regarding the organisation and its respective staff members, critical outcomes to ensure successful implementation at the site need to be achieved, such as organisational readiness to promote an enabling working environment for PSWs in which their expertise is recognised. This entails the preparedness of the mental health services, including for example leadership support, institutional development and resource allocation to integrate and support PSWs working in the institution. During this phase of initial implementation, transparent integration of peer support into the local network of mental health services and enhanced ownership and sustained commitment from the public, stakeholders, and funders need to be achieved. The processes up to this point can be initially piloted at a small scale to inform the further adaptation of the intervention as needed, to be repeated afterwards at a larger scale.

Once the initial implementation of peer support has been successful, several outcomes need to be achieved to ensure sustainability in the long-run. One important pre-condition for long-term sustainability is the availability of evidence of the intervention’s effectiveness in improving outcomes of SUs and PSWs. The continuous engagement of the public, funders, and other relevant stakeholders throughout the implementation phase will help to set the groundwork and inform plans for the long-term integration of peer support into services. With time a cultural shift within systems through the distinctive contribution of PSWs working within an organisation and working with clients in their communities will be achieved. This will help to foster reduced in-system stigma of mental ill-health and lead to PSWs becoming advocates for mental health awareness in the community. Successful initial implementation at one site may then also lead to a more diverse offer, for example through scale up to other institutions, cities or countries.

Various indicators were defined to check whether outcomes were achieved. Data were collected against these indicators either quantitatively (via routine monitoring and evaluation) or qualitatively (through focus group discussions).

#### Interventions

Several different interventions need to be carried out in order to achieve the outcomes described above. In the preparatory phase, these consist mainly of activities revolving around spreading and gathering information through piloting, focus group discussions, consultation with stakeholders, introductory presentations, and recruitment. Once the preparatory phase is concluded, then capacity-building activities (training PSWs, organisational readiness workshops, setting up a support system), as well as the involvement of SUs and wider public can commence (delivering peer support, networking, public engagement). The last intervention that is possible to be conducted during the official duration of the UPSIDES study is the conduct of the RCT which will help to achieve long-term outcomes for sustainable implementation. Generating gold-standard evidence of the effectiveness of peer support will help to advocate for long-term integration of peer support into mental health services.

#### Assumptions

There are several essential assumptions underlying the pathway of change at a given site. These include: sufficient demand for peer support as well as readiness and desire for change in the target population of service users; appropriate mind-set of the prospective PSWs (motivation, perseverance and readiness); the diversity of PSW team reflects the diversity of personal backgrounds among service users; a critical mass of staff members are willing to engage with PSWs; support from other relevant stakeholders; and PSWs are working in an organisation where they can work in teams with other staff members.

#### Rationales

Rationales underlying the pathway of change are based on evidence generated from UPSIDES and other relevant studies of peer support. The rationales behind the ToC’s elements of organisational readiness, collaboration and local adaptations were derived from insights from UPSIDES focus group discussions with key stakeholders and mental health staff members which were especially important at the early phases. Further insights of previous studies on peer support informed the rationales behind the importance of a clear role description and the impact of peer support on different outcome variables.

### Site-specific features and adaptations

A feature that was unique to the maps from Be’er Sheva, Hamburg, and Ulm was a stronger focus on collaboration with other mental health service providers or teams, whereas stakeholders at sites in Pune, Kampala and Dar es Salaam focused more on community integration. Further, workshop participants in Kampala and Pune discussed the form of reimbursement and its consequences, financial empowerment of PSWs through peer support work and other possible income generating activities apart from peer support work, whereas discussions in Be’er Sheva, Hamburg, and Ulm revolved more around the PSWs’ role as employees and professionalization rather than financial reimbursement.

Further, we saw differences in the levels of sustainability addressed in the workshops. The discussions in Ulm, Dar es Salaam and Pune revolved more around the integration in the institution and efforts to build a network for PSWs. The site-specific ToC maps in Butabika, Be’er Sheva and Hamburg also included more elements referring to sustainability, for example long-term employment, integration in routine mental health care or scale up to other institutions.

## Discussion

As a result of this study, a cross-site ToC map was developed which divides the implementation of the UPSIDES peer support intervention into three main stages (preparation, implementation, and sustainability), and identifies three levels of actors (individuals—i.e., service users and peer support workers—organisations, and the public). The cross-site ToC is a representation of shared intermediate outcomes, interventions and actors involved in the implementation of UPSIDES peer support across various study sites in diverse settings.

### Key findings and interpretation

The cross-site impact statement that was formulated (“Improved social inclusion and reduced stigma of people with severe mental health conditions through systemic and cultural change”) reflects elements of the Conceptual framework of IMpacts of Recovery Innovations (IMRI), as it describes the intervention’s impact on future ways of being, thinking, interacting and operating in mental health systems and communities towards people with mental health conditions [26].

The UPSIDES cross-site ToC describes key factors and processes for the implementation of mental health peer support to achieve this impact across a range of high-, middle-, and low-income settings. Considering that peer support is an extremely flexible intervention that can and should be adapted to local circumstances [7], there was a need for stringent monitoring and evaluation (M&E) of the implementation processes across sites throughout the project implementation phase. The ToC approach helped to derive concrete steps for local implementation, to adapt the implementation to the different settings across study sites, and to develop protocols for sites’ M&E.

The main differences in local implementation as reflected in the site-specific maps can be explained by several factors, i.e., resource setting, different levels of readiness for implementing peer support, stigmatisation of people with mental health conditions, and strength of hierarchy within mental health systems. Workshops at sites located in HIC focused heavily around collaboration with other mental health service providers or teams, whereas stakeholders at sites in LMIC focused more on integrating peer support into the local community. This is in line with other research in the field that highlights the importance of the community when addressing mental health care in LMICs [27]. Further, the form of reimbursement and its consequences was an important topic for sites in LMIC. The financial situation of PSWs in LMICs might be more critical than that of their colleagues in HICs [28], most of whom receive at least some sort of social benefits [29]. Often in LMIC healthcare and other essential needs are paid for out-of-pocket [30]. Thus, for those who have lived experience of a mental health condition, reimbursement for peer support work can help to meet basic needs and enable longer-term improvements [31]. Taken together, it is unsurprising that the tangible community and monetary aspects of peer support work were more prominent in discussions in lower-income settings. The importance of resource availability was also reflected in the qualitative UPSIDES studies with stakeholders in LMICs [22]. By comparison, the discussions in HIC revolved more around the PSW’s role as an employee and their professionalization. PSWs in LMICs may be more warmly welcomed into severely understaffed mental health services, while in HICs, they may not be as readily accepted or regarded as competitors by other mental health professionals [32, 33]. This discourse on professionalization is very much in accord with other research on lived experience implementation and relationships in HICs [34]. Further, the topic of collaboration and professionalization is more important in the integrated care system common in many HICs which attempts to coordinate across a wider range of services including those outside the health care system [35].

We also noticed some differences between the site-specific maps based on the level of previous experience with peer support and subsequently different levels of readiness at the site, as described in a previous UPSIDES publication on barriers and facilitators for implementation of peer support [7]. The discussions at sites with lower readiness for implementation (Ulm and Dar es Salaam) revolved more around integration into the institution and efforts to build a network for PSWs. The ToC maps of the sites with higher levels of readiness and several years of previous experience with implementing mental health peer support (Be’er Sheva, Butabika, Hamburg) through other projects and initiatives [36–38] included more elements referring to sustainability, for example long-term employment, integration into routine mental health care, or scale-up to other institutions.

Varying levels of stigmatisation of mental health and strength of hierarchy within the mental health system in the different workshop sites were an important challenge when planning the overall conduct of the workshops. Service users and caregivers often face high levels of stigma, leading to difficulties in actively engaging these two crucial types of stakeholders in research [8, 39]. Further, strong hierarchical structures in the provision of mental health services also may add to challenges when involving different groups of stakeholders in a project [8, 10]. In settings with high levels of stigmatization and strong hierarchies within the mental health institution, it was helpful to hold separate workshops with each stakeholder group, e.g., one with nurses, one with hospital board members, and one with carers and service user representatives, then synthesising all inputs into one ToC map afterwards. This helped participants to feel more confident and promoted their active participation during the workshops. Previous ToC research has reported similar findings regarding methods of engaging different stakeholder groups [10].

### Implications

As a next step, the UPSIDES cross-site ToC map (particularly the indicators) will be used to guide further theory-driven analyses of the UPSIDES study regarding the impact of factors related to implementation on effectiveness, i. e. as a conceptual framework indicating moderators and mediators of effect on various outcomes guiding the process evaluation. By highlighting possible pathways of change on several levels (service users, peer support workers, organisations) it will also help to understand patterns and differences in effects across sites. For example, qualitative comparative analysis can be used to provide an integrated analysis of data as shown in previous ToC-driven evaluations [40].

Another use of this research on ToC is in facilitating evaluation of other peer support programmes. Our ToC may be instructive to other studies seeking to carry out routine M&E, as well as process evaluation, by suggesting indicators that may be important to assess.

The study also aids to identify knowledge gaps in the field and facilitates new concepts and assumptions to emerge. For example, the prominent influence of organisational readiness, the cooperation with other staff members and the peer support workers’ involvement in the community are important features of peer support, with need for more research in this area.

### Strengths and limitations

Due to the pandemic, most workshops could not be held in-person in 2020 and 2021 after the initial ToC workshop. As a result, many site-specific maps could not be refined with stakeholders via in-person meetings. Therefore, the HIC sites in Israel and Germany conducted online meetings to discuss the current drafts of their site-specific map. Online workshops were feasible; however, in-person meetings were preferred since discussions flowed more easily and collective working with sticky notes and flipcharts promoted creativity and active involvement of all participants. Online workshops via video conferences were not feasible in the LMIC sites. Despite these challenges, the study at hand is one of few [12] which has revised and updated some of their site specific maps and the cross-site ToC map over the course of the study to incorporate important contextual changes (i.e., the pandemic) as well as key learnings from the implementation phase into the final map.

Another limitation is the considerable variation in interest and participation in the ToC workshops across the sites (e.g. 4 participants in PU vs. 74 participants in BGU). This can be partly explained by the difficulties with conducting follow-up workshops as result of the COVID-19 pandemic, as described above. Through ongoing expert consultation with colleagues from all study sites during the development of the cross-site ToC, we have tried to mitigate the over- and under-representation of stakeholders from different study sites.

No sociodemographic data of workshop participants apart from their professional background was available for this study which limits further interpretation of the workshop results in a broader socio-cultural context. For the purposes of the UPSIDES study, this did not impact successful use of the ToC for implementation and evaluation.

What makes this study stand out from previous research with ToC in several sites is that the cross-site map was derived from site-specific maps and not vice-versa, as is often the case [10]. This may represent both a limitation and a strength. When merging site-specific maps into one cross-site map, some details must inevitably be obscured for the map to concisely represent general processes of implementation. However, the strength of this approach is that sites develop their site-specific ToCs towards a shared goal unbiased by a cross-site map, allowing the sites to develop their ToCs independently and thereby fostering creativity and unique ideas. Having separate site-specific ToCs also helped to keep track of the differences in implementation that could plausibly affect site-specific outcomes, which can subsequently be explored through secondary analyses.

Another aspect that makes this ToC research stand out from others is that we were working with different languages mostly in non-Anglophone HICs and LMICs with a mixture of English and other local languages. The synthesis of maps of multilingual origin was challenging and there was a very real risk of losing information in translation. We have mitigated this risk by sticking to a pre-defined translation methodology and by conducting several feedback rounds with representatives of all sites.

Ultimately, all sites managed to include different stakeholder groups in the ToC process, leading to multi-faceted representation in all ToC maps. In addition, the concept of an explicitly participatory ToC that actively involves stakeholders from the outset differs from ToCs that were developed in other ways. Another strength of this study is that the ToC was developed as part of a research project with standardised operating procedures for data collection and reporting across all sites. Consequently, data collected against the indicators assigned to each outcome are available for further analyses across all sites.

## Conclusions

The development of a cross-site ToC in UPSIDES helped to crystallise core elements of implementing and evaluating a peer support intervention across different socio-cultural, systemic and income-level settings. Through this participatory approach, stakeholders and study teams worked together to identify common approaches to implementation and measurement across all sites, while also determining local specifics and challenges that necessitate careful adaptation to the local setting. Successful local implementation while maintaining fidelity to some core elements is an essential precondition for a multi-site evaluation to be able to draw meaningful conclusions. The insights provided by the cross-site ToC will be used in the further evaluation of the UPSIDES peer support intervention. We believe that the ToC we have created also holds relevance for other research and policy initiatives aiming to implement peer support in various settings. Further, our ToC approach can inform and guide other multinational collaborations, contributing to the advancement of implementing evidence-based complex interventions in the realm of global mental health.

### Supplementary Information

**Supplementary Material 1. **

**Supplementary Material 2. **

## Data Availability

Meeting minutes generated during the ToC workshops are not publicly available, as they contain information that could compromise the privacy of research participants.

## Abbreviations

HIC: High Income Country
LMIC: Low- and Middle-Income Country
ToC: Theory of Change
UPSIDES: Using Peer Support In Developing Empowering mental health Services
UULM: Department of Psychiatry II, Ulm University, Ulm, Germany (study site)
UKE: Department of Psychiatry, University Medical Center Hamburg-Eppendorf, Hamburg, Germany (study site)
BU: Butabika National Referral Hospital, Kampala, Uganda (study site)
DS: Dar es Salaam, Tanzania (study site)
BGU: Department of Social Work, Ben Gurion University of the Negev, Be'er Sheva, Israel (study site)
PU: Centre for Mental Health Law and Policy, Indian Law Society, Pune, India (study site)
LAB: Local Advisory Board
PSW: Peer support worker
SU: Service user
M&E: Monitoring and Evaluation
