# Supplementary material for: Implementation of peer support for people with severe mental health conditions in high-, middle- and low-income-countries: a theory of change approach
Source: BMC Health Serv Res. 2024 Apr 18;24:480. doi: 10.1186/s12913-024-10990-5 (PMC11027518; doi:10.1186/s12913-024-10990-5)
Supplement: Supplementary file 1 — Supplementary Material 1. [file 12913_2024_10990_MOESM1_ESM.pdf]

Supplementary File 1

File format: .pdf

Title: ToC guidance

Description: Facilitators' guidance on ToC workshops as provided in the Implementation Plan

Excerpted from:
Nakku, J.; Ryan, G.; Aslam, R.; Baillie, D.; Adler Ben-Dor, I.; Charles, A. et al. (2023): UPSIDES Cross-Site Implementation Manual: Generic Field Version. UPSIDES Project. Available online via: https://www.upsides.org/multilingual-area/output/#32Manuals

Appendix 6 - Theory of Change

Introduction to Theory of Change

What is a Theory of Change?

Theory of Change is an approach to designing, describing and evaluating complex interventions that is increasingly being used in concert with the Medical Research Council's Framework for Complex Interventions in order to help structure the research process.

A Theory of Change is often depicted as a *map* describing all the different components of a complex intervention, how they relate to one another, and how they are expected to work. A detailed Theory of Change map should include the components outlined in the below table (adapted from DeSilva et al. 2014 and Ryan et al. 2018). An example map is also provided at the end of this document.

Core components of a Theory of Change map

Terminology	Definitions	Examples
**Outcomes**<br>(i.e. "Pre-conditions" or "Milestones")		
‣ Short-term, Intermediate	The intended results of the interventions. Things that don't exist now, but need to exist in order for the logical causal pathway not to be broken.	Change in knowledge, attitudes and skills of lay health workers to enable them to successfully deliver talking therapy.
‣ Long-term	The final outcome the program is able to change on its own.	Reduced prevalence of CMDs in the population receiving talking therapy.
‣ Ultimate<br>(i.e. "Impact" or "Goal")	The real-world change you are trying to affect.	Reduced prevalence of CMDs among survivors of humanitarian crises.
**Interventions**<br>(i.e. "Strategies")	The different components of the complex intervention.	Training of lay workers on the delivery of talking therapy.
**Indicators**	Things you can measure and document to determine whether you are making progress towards, or have achieved, each outcome.	Reduction in symptom severity for CMDs.
**Rationale**	Key beliefs that underlie why one outcome… [leads to] the next, and why you must do certain activities to produce the desired outcome.	Humanitarian responders need to be educated about signs and symptoms of CMDs in order for CMDs to be detected during crises.
**Assumptions**	An external condition beyond the control of the project that must exist for the outcome to be achieved.	Task-sharing with lay workers is socially and politically acceptable.

Why have a Theory of Change?

There are many benefits to having a Theory of Change which have been described in the academic literature. For UPSIDES in particular, there are three main reasons why it is important:

- *To better understand similarities and differences across UPSIDES sites:* Peer support is going to be implemented in slightly different ways across each of the UPSIDES sites. Theory of Change maps are useful visual tools to help us compare and contrast how peer support will look at each site.
- *To inform methods for process evaluation at each site:* Because UPSIDES is an implementation research project, it is particularly important that we capture data on processes, not just outcomes. Theory of Change can help us think through the processes that we need to evaluate and assign indicators to them.
- *To ensure meaningful involvement of key stakeholders:* The process of developing a Theory of Change can help to improve stakeholders' understanding and buy-in. The resulting map can also be a useful communications tool to explain to stakeholders what is being done at each site and how.

Creating a Theory of Change

How do I conduct a Theory of Change workshop?

The best way to start developing a Theory of Change map is with a *Theory of Change workshop*. This is where key stakeholders are gathered in a room to first consider the ultimate impact that peer support aims to achieve, and then work backward to identify the key steps necessary to bring about this change. Often, a Theory of Change workshop will consist of 5-15 stakeholders with complementary expertise (e.g. service providers, service users, caregivers, researchers, etc.) led by a facilitator. The facilitator will ask a number of questions to the group, such as:

- *"What is the impact or change in the real world that we want to achieve?"*
- *"What outcomes are needed to produce this impact?"*
- *"What interventions are needed to produce these outcomes?"*

As stakeholders' respond to these questions, the facilitator summarises their responses on sticky-notes or sheets of loose paper, and continually arranges and rearranges them on a wall, a large table or even the floor, to give a sense of the sequence of steps on the map.

At the end of the workshop, the facilitator typically takes photographs of the initial map that has been produced and may even keep the original sticky-notes or pieces of paper.

When should I conduct a Theory of Change workshop?

As many key stakeholders are already involved in your Local Advisory Boards, we recommend that you use one of your earliest *Local Advisory board Meetings* to hold a Theory of Change workshop. However, you are very welcome to conduct additional Theory of Change workshops with other relevant stakeholders as you see fit.

What should be the output of my workshop?

It is very rare that a single Theory of Change workshop is sufficient to produce a complete map. The facilitator should take some time soon after the workshop to revisit what was done in the workshop

and try to refine and develop it further, typically using Powerpoint. The *Powerpoint slide* can then be fed back to the stakeholders who participated in the workshop and also shared with others who didn't participate in the workshop, for further input.

What are the next steps after the workshop?

Once you have a draft Theory of Change map ready to share in Powerpoint, please send it to the *Work Package 5 leads*, who can give advice on how to refine and develop it further. The aim will be to have a complete, revised draft ready in time for your next Local Advisory Board meeting, so that your stakeholders have an opportunity to review it.

The Theory of Change map will be *a living document* that is revised throughout the life of the project as it evolves, as you encounter challenges and new solutions to overcome those challenges, and as you collect data. We suggest revisiting the Theory of Change map at each Local Advisory Board meeting. The Theory of Change map will only be finalized after the implementation and evaluation phases of UPSIDES have concluded.

Where can I learn more about Theory of Change?

The Mental Health Innovation Network also has an online Theory of Change Toolkit including a practical guide to creating a Theory of Change map. This is particularly useful for the workshop facilitator to review.

If you're interested in learning more about Theory of Change from an academic perspective or seeing how it has been used in previous multi-country research projects and/or research evaluations of mental health interventions in low- and middle-income countries, here are some additional references:

- Asher et al. (2015). Development of a Community-Based Rehabilitation Intervention for People with Schizophrenia in Ethiopia. *PLoS One*, 10(11):e0143572.
- Breuer et al. (2016). Planning and evaluating mental health services in low- and middle-income countries using theory of change. *BJ Psych*, 298(s56):s55-s62.
- Breuer et al. (2018). Theory of change for complex mental health interventions: 10 lessons from the programme for improving mental health care. *Glob Ment Health*, 5:e24.
- Chibanda et al. (2016). Using a theory driven approach to develop and evaluate a complex mental health intervention: the friendship bench project in Zimbabwe. *Int J Ment Health Syst*, 10(16).
- DeSilva et al. (2014). Theory of Change: a theory-driven approach to enhance the Medical Research Council's framework for complex interventions. *Trials*, 15:267.
- Hailermariam et al. (2015). Developing a mental health care plan in a low resource setting: the theory of change approach. *BMC Health Serv Res*, 15:429.
