# Supplementary material for: Implementation of peer support for people with severe mental health conditions in high-, middle- and low-income-countries: a theory of change approach
Source: BMC Health Serv Res. 2024 Apr 18;24:480. doi: 10.1186/s12913-024-10990-5 (PMC11027518; doi:10.1186/s12913-024-10990-5)
Supplement: Supplementary file 2 — Supplementary Material 2. [file 12913_2024_10990_MOESM2_ESM.pdf]

Supplementary file 2

File format: .pdf

Title of data: Site-specific ToC maps

Description: Supplementary file 2 contains the drafts of the site-specific Theory of Change maps of all UPSIDES study sites and a table with an overview of commonalities and differences between all drafts.

Table 1 in Supplementary File 2. Commonalities and differences in drafts of ToC maps.

Commonalities	Differences and unique elements in each study site					
	UULM	UKE	BU	DS	BGU	PU
Collaboration and networking in all drafts	Mainly within institution and with other mental health providers	Mainly with other mental health providers	Within institution and with the community	Collaboration with mental health providers and with community	Mainly within and with other mental health providers	Focus on collaboration with the community
Training and stakeholder engagement as interventions in all drafts	Other interventions mainly pre- and during trial	High number of post-trial interventions	Several other pre-, during and post-trial interventions	Other interventions only pre- and during trial	Several other pre-, during and post-trial interventions	No other interventions
Finances, employment or reimbursement in 5 of 6 drafts	Mentions financial compensation of PSWs, focus on role and integration of PSWs as staff members	Mentions budget and PSWs as staff members	Mentions reimbursement of PSWs and income-generating activities	No mention of finances or employment	Mentions employment and professionalisation of PSWs	Mentions financial empowerment of PSWs
Organisational readiness in 5 of 6 drafts	Mentioned in two outcomes: 1) staff members ready for PS and 2) overcome staff members' timidity and reserve	Described as social inclusion on an institutional level	Described as preparedness of staff to create an enabling environment for PSW	Described as awareness of staff before integration of PSWs in hospital	Organisational Readiness explicitly mentioned as outcome	No mention of organisational readiness
Different phases of the project (pre-, during and post-trial) mentioned in 5 of 6 drafts	Focus on elements pre- and during trial, mentions scale-up after trial briefly	No pre-trial outcomes, few pre-trial interventions, focus on sustainability post-trial	All phases of the project, long-term integration in services plays prominent role	Focus on activities pre-trial and during the trial	All phases of the project, sustainability post-trial explicitly mentioned	No differentiation between phases
Causal links between ToC elements in 4 of 6 drafts	No causal links	Causal links indicated in draft	Causal links indicated in draft	Causal links indicated in draft	Causal links indicated in draft	No causal links
Final impact after ceiling of accountability defined in 3 of 6 drafts	Peer support succesfully implemented	Enhance the social inclusion of people with mental health problems and decrease stigmatization of men-tal health problems	PSW is integrated into routine mental health care in Uganda & other countries	Final impact "Recovery" not further elaborated	No final impact statement	No final impact statement
Assumptions, indicators and rationales described in 5 of 6 drafts	Assumptions, indicators and rationales described	Assumptions, indicators and rationales described	None available	Assumptions, indicators and rationales described	Rationales described	Assumptions, indicators and rationales described

Note. PSW = peer support worker; PS = peer support; UULM = Ulm, Germany; UKE = Hamburg, Germany; BU = Kampala, Uganda; DS = Dar es Salaam, Tanzania; BGU = Be'er Sheva, Israel; PU = Pune, India.

UPSIDES

ToC-Map English, Version 1.2 (04/12/2018), Ulm

ulm university universität uulm

ToC UULM

PS succesfully implemented

Public

- clarification of purpose & positioning of PS within mental health system by UPSIDES team and LAB
- UPSIDES not as competition to existing structures! → Appreciate other services, then demarcate from other services → UPSIDES should not add to complexity
- Define add-on of UPSIDES within existing structures!
- **Clear positioning of PS in the local mental health system**
  - Current obstacle: multitude/diversity of offers → Complexity, lack of clarity & lack of inter-communication
- **transparency**
- Low-threshold access → define the hurdles: 1. location (e.g.city better than hospital), 2. health insurance applications, 3. ...)
- Networking (also as a task of the PSW): 1. Within the clinic (e.g. HT and wards) 2. Outside (e.g. schools)
- **Establish PS network**
- **Professionals must recognise PSW as „experience experts“ (recognise & understand the different apporach of the PSW)**

(other) facilities / institutions

- Well attended (info-) events & low-threshold publication / "advertisement"
- Presentation on PS in other countries (e.g. invite BU / DS/ BGU / PU)
- **Other people (not affected, other occupational groups) know about it. → Knowledge is missing, PSW need perseverance**
  - Potential SUs will be referred to PSW
- **(other) points of PSW presence**
  - GPs and resident psychiatrists
  - schools
  - Religious insititutions
- Provide office space (with low threshold access)
- Provide supervision
- Provide financial compensation
- **PS is being offered**

Service users

- Define SU (e.g. low threshold, ICD-10 diagnosis)
- **Affected persons in the region**
  - Affected persons with desire for change
- **Affected persons know about PS (& know that it could help them)**
- **SU seek PS offer**
- **Matching dyad (SU ↔ PSW)**
- **SU use PS offer**
  - Duration, frequency

Peer support workers

- Define PSW
- **Affected persons want to work as PSW**
  - Passion & motivation
  - perseverance
- PSW training (enable on-site training & financing)
- prepare peers for future tasks (Ex-In only peer-oriented)
- Learning to understand one's own role (distance from one's own medical history to SU's history)
- **PSWs are trained**
- **PSW know their role and tasks (individually and facility specific)**
  - Rationale LAB / TOC-1: PSWs also want "double-sided handrails"; total flexibility/freedom may be too demanding
- **PSWs receive supervision (UPSIDES team)**
- Team building, Mutual Support Groups
- **PSW teams (no lone warriors)**
  - Rationale from FG1.1_1.2: Works better, more "meaning", more support if necessary
  - Rationale from LAB / TOC-1: Affected without desire to change in day care centres → other PSW colleagues give energy and empowerment

staff members (of participating facilities)

- staff training (e.g. how to talk to a PSW)
- **Staff members ready for PS**
- **Overcome staff members‘ timidity and reserve**
- **PSWs integrated into teams (not subordinate) → own voice/position of the PSWs**
  - Amount of team meetings with a PSW
  - There are existing teams in the facility

Providers (of participating facilities)*

- Define (role of) providers
- Recruitment of (further) participating facilities (e.g. parishes)
- **Participating facilities**
  - Willing to invest money
- Possibility for institutions to adapt the role of the PSWs

* Parishes and other religious communities, social psychiatric services, clinics and hospitals, charitable services, general practicioners, resident psychiatrists, municipal counselling agencies, schools

key

Intermediate outcome	(blue box)	Assumption	(grey box)
Intervention	(orange ellipse)	Rationale	(yellow hexagon)
Indicator	(red flag)	Impact	(blue filled box)

Abbreviations:
PSW – Peer Support Worker
PS – Peer Support
SU – Service User
LAB – Local Advisory Board
HT – Home Treatment

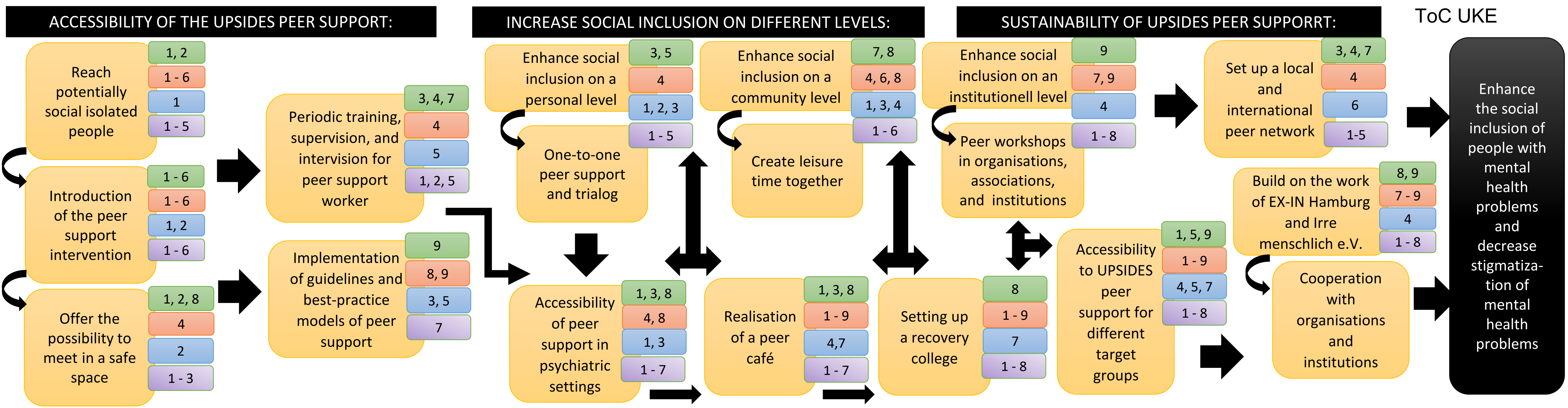

Interventions

How can we reach the preassumtions?

(1) Promotion of UPSIDES peer support
(2) Enhance the UPSIDES online and social media performance
(3) Enlarge the UPSIDES team via peer suppert worker and train-the-trainer courses
(4) Additional training for UPSIDES peer-advocates
(5) Translate all UPSIDES materials to easy-to-understand language
(6) Realise public relations, media presence and awareness capaigns
(7) Periodic get-together event for UPSIDES peers
(8) Periodic public events in various contexts
(9) More research and peer-led research

Indicators

How can we evaluate, if the interventions are succesful?

(1) Using differnt statistics
(2) Record, if the social media accounts are active
(3) Number of participants
(4) Assesing the participants feedback
(5) Record, who is reached and how
(6) Assess the request for peer support in various settings
(7) Assess changes in the roles of mental health staff members
(8) Assess social inclusion
(9) Assess changes in the attitutes towards mental health problems

Rationals

Why do we think, that the interventions will be succesful?

(1) The persons are reached, where it is important for them.
(2) Peer support is a low threshold intervention.
(3) Peer support has been proved to be effective.
(4) Peer support in various contexts makes the topic of social exclusion visuable and enables to talk about it.
(5) Sufficiant request for peer support and peer training in Hamburg.
(6) Intervision and networking are key factors in the implementation of peer supprt.
(7) Offers an opportunity to usual care.

Assumptions

Which additional factors have an influence on the interventions?

(1) Sufficiant bugjet
(2) Sufficiant staff
(3) Premises and rooms
(4) Data protection regulations
(5) Sufficiant request
(6) Interest and cooperation of different stakeholders and organisations
(7) Political developments
(8) Social change

ToC BU

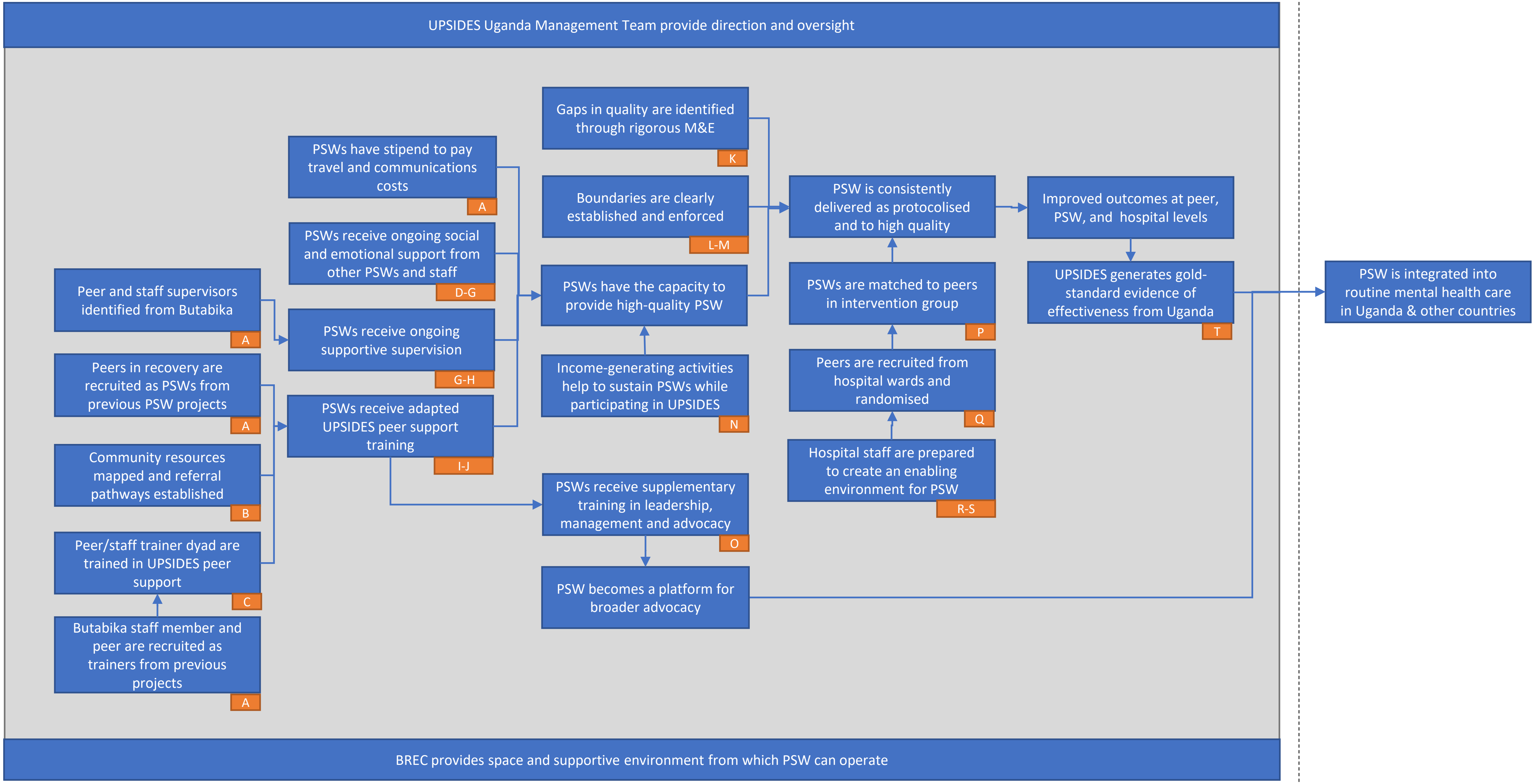
UPSIDES Uganda Management Team provide direction and oversight
Gaps in quality are identified through rigorous M&E
K
PSWs have stipend to pay travel and communications costs
A
Boundaries are clearly established and enforced
L-M
PSW is consistently delivered as protocolised and to high quality
Improved outcomes at peer, PSW, and hospital levels
PSWs receive ongoing social and emotional support from other PSWs and staff
D-G
Peer and staff supervisors identified from Butabika
A
PSWs have the capacity to provide high-quality PSW
PSWs are matched to peers in intervention group
P
UPSIDES generates gold-standard evidence of effectiveness from Uganda
T
PSW is integrated into routine mental health care in Uganda & other countries
PSWs receive ongoing supportive supervision
G-H
Peers in recovery are recruited as PSWs from previous PSW projects
A
Income-generating activities help to sustain PSWs while participating in UPSIDES
N
Peers are recruited from hospital wards and randomised
Q
PSWs receive adapted UPSIDES peer support training
I-J
Community resources mapped and referral pathways established
B
Hospital staff are prepared to create an enabling environment for PSW
R-S
PSWs receive supplementary training in leadership, management and advocacy
O
Peer/staff trainer dyad are trained in UPSIDES peer support
C
PSW becomes a platform for broader advocacy
Butabika staff member and peer are recruited as trainers from previous projects
A
BREC provides space and supportive environment from which PSW can operate

Interventions/Activities

A. Define selection criteria, job descriptions and compensation and/or stipends for each role.
B. Consult LAB members and other stakeholders to identify relevant community resources and contacts, including both secular and non-secular leaders and organisations
C. Participate in UPSIDES cross-site training in Tanzania
D. Establish PSW "Buddy" System
E. Assign "Wellness Officer" from among hospital staff
F. Organise social and wellness activities (e.g. film club, yoga) in which PSWs can take part
G. Organise mutual support group meetings for peer-to-peer and group supervision
H. Organise regular one-to-one supervision and "shadow visits"
I. Adapt UPSIDES training manual and materials for use in Uganda
J. Organise training including competency assessment
K. Establish system for routine monitoring
L. Establish code of conduct
M. Train PSWs in MAPA for de-escalation
N. Establish income-generating activity, e.g. beading
O. Develop and organise supplementary training
P. Develop criteria and procedure for "mapping"
Q. Develop system for recruitment as per study protocol
R. Carry out organisational readiness workshops
S. Regularly disseminate UPSIDES newsletter to staff
T. Evidence is published and disseminated to academic and non-academic audiences, including policy-makers

TOC Map for UPSIDES, Tanzania

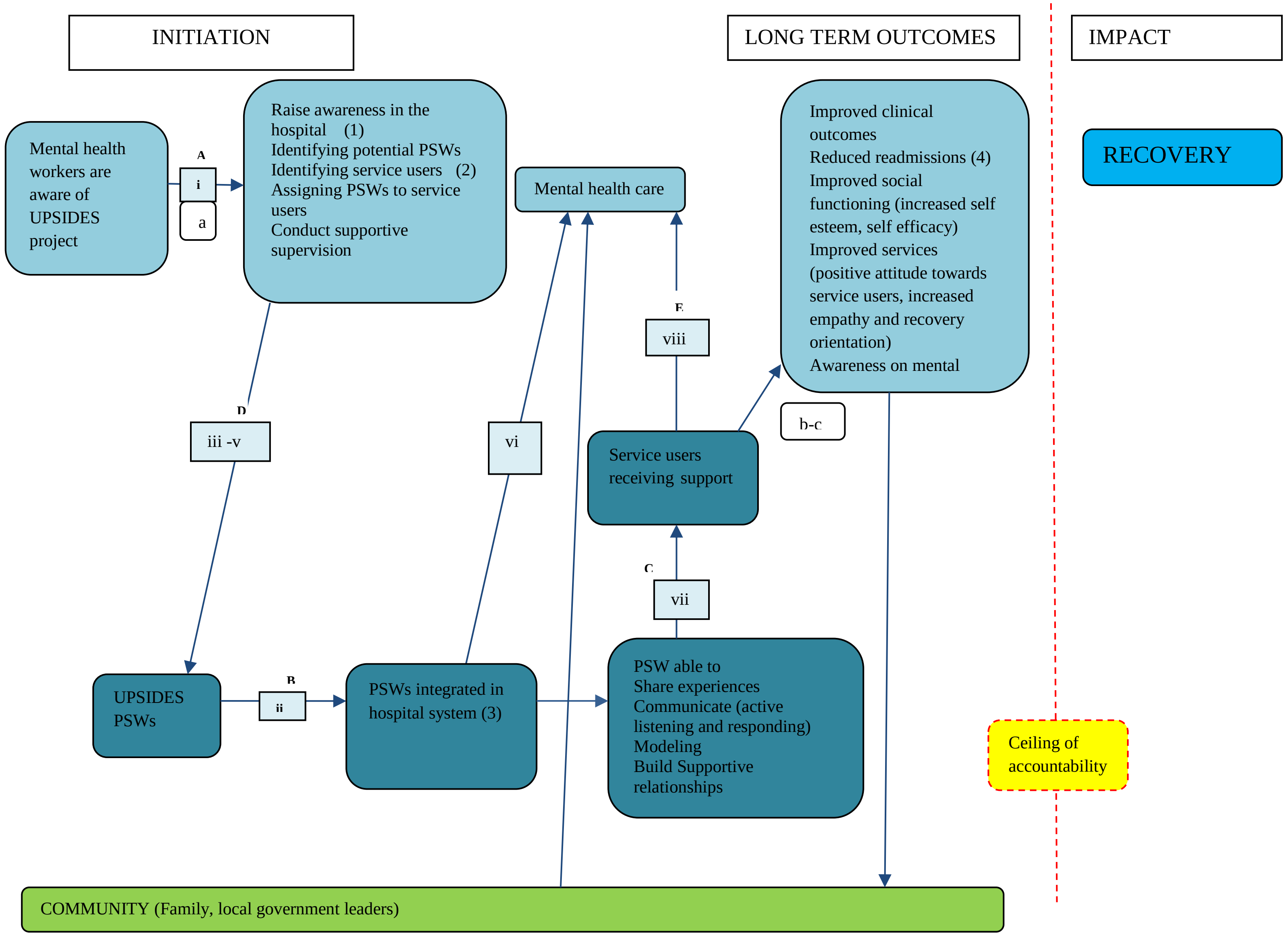

- Mental health Workers
- PSWs
- i Intervention
- A -Assumption
- a Rationale
- 1- Indicators

ASSUMPTIONS

A. Mental Health Workers are willing to engage with the project and supervise PSWS.
B. PSWs with potential qualities consent and undergo training.
C. Service users are willing to receive peer support from PSWs
D. PSWs continually supervised and helped with challenges.
E. Mental health workers are willing and able to accept referrals from PSW.

INTERVENTIONS

i. Conducting organization readiness workshop
ii. Training of PSWs
iii. Mental health workers screen potential service users and PSWs.
iv. Service users are assigned/referred to PSWs
v. UPSIDES research team conducts supervision with mental health workers and PSWs.
vi. Relapsed PSWs receive specialist care
vii. Peers conduct a minimum of 3 visits weekly to each assigned service user.
viii. Relapsed service users receive specialist care

RATIONALE

a. Health care providers should be aware of the project so as to assist PSWs.
b. Research shows that PSWs are better at reducing inpatient services and improve relationships with health care provider's engagement with care and variety of recovery outcomes.
c. Mentally ill patients are seen treated and return to their social functions.

INDICATORS

1. Mental health workers at Muhimbili National Hospital are aware of the project.
2. Mental health workers have competencies to screen service users.
3. PSWs have appropriate skills to support service users after the training.
4. Reduced CMD symptoms and number of admissions

Theory of change in order to develop, evaluate and implement the peer support work intervention in mental health service in Israel

ToC BGU

Participatory approach by bringing together a range of stakeholders

Identifying PSW developmental needs √

FG 1+2: **14 stakeholders** - PSW's, multidisciplinary MH directors, practitioners which employ PSW in Israel. *Over the past decade, diverse peer roles were developed in Israeli mental health system.* Participants related *to Lack of clear job definitions for PSW's role in Israel; Uncertainty about peer providers' occupational identity.* Staff and PSW were unsure when and how much peers should share their lived-experience. Participants pointed to a combination of **unique-peer** and **professional** skills which are desired for peer provider.

Identifying organizational criteria for optimal implementation of PSW √

FG 1+2: 14 stakeholders -Preliminary analyses shed light on challenges to the sustainable implementation of peer support in MH services: *Participants emphasized that multi-level conditions are needed in order to ensure fulfillment of peer provider roles.* Peer support begins **'top-down':** *A need for policy support and organizational readiness.* Peer support continues '**bottom-up':** A need for peer providers' and service users' readiness for change.

Deep Interviews with stakeholders and local advisory emphasized the need to further support the occupational development and career track of peer support workers by **striving for a deeper understanding of the concept of peer roles, developing training and supervision** whilst keeping to a shared learning, building mutuality, and shared meaning making framework. √

Developing proper UPSIDES Manual training and supervision √

FG3: All the participants were local stakeholders with experience of either: employing peer support workers; working as peer support workers ; peer trainers; service users. The participants mentioned and described additional modules recommended to be developed in the manual training and changes need to be **made and cultural adaptation.**

Piloting phase: √

Developing a TOC must be a continual process of reflection and adaptation as barriers to implementation arise and new evidence comes to light, requiring pathways to be changed and strengthened.

This was an important learning for us, to see the value in developing the UPISDES **intervention into a small-group intervention – as an add** on that will occur with new peer-clients that peer support workers have not been working with before. This will make it easier to use their live experience more explicitly with them. √

Organizational readiness √

We find that **organizational readiness** needs to be denser already **at early stages of implementation**. Therefore, before the second training has started, we conducted 4 meetings to introduce the project and PSW with directors and team members .

Second UPSIDES training √

We considered diverse questions, e.g-: how many PSW need to be trained in order to deliver the intervention as intended, a special attention was given to the **ripeness of the person** to use their lived experience, their **intrinsic motivation** for this role and the **set conditions** for them to successfully complete training.

Professional PSW working permanently in services √

An evaluation based on TOC will require a number of different methods to capture all of the indicators- **RCT and qualitative sub study**

Successful and sustainable peer support intervention √

Ddescription of the intervention which can be disseminated to a variety of audiences, providing information on the components of the intervention that need to be adapted for use in other settings.
2 FG should be conducted on 22/12/2019 with Participants with **relevant organizational knowledge regarding the implementation of UPSIDES peer support** √

ToC PU

THEORY OF CHANGE

OUTCOMES

- Social inclusion
- Empowerement
  - emotionally
  - Financially

INTERVENTIONS

Stakeholders

* Hospital
* NGOs
* Govt. agencies (ex. employment agency)

Activities

* PSV training
* Develop vocational skills.

INDICATORS

PSVs getting into the community.

RATIONALE

* Social Inclusion
* To reduce stigma
* To fight stigma
* More involvement
* Employment.

ASSUMPTION

* Support from all stakeholders
* Readiness of PSVs to go back into the community
* Community accepting persons with SMD.
